# Acknowledgment to the Reviewers of *Medicina* in 2022

**DOI:** 10.3390/medicina59020194

**Published:** 2023-01-18

**Authors:** Medicina Editorial Office

**Affiliations:** MDPI AG, St. Alban-Anlage 66, 4052 Basel, Switzerland

High-quality academic publishing is built on rigorous peer review. *Medicina* was able to uphold its high standards for published papers due to the outstanding efforts of our reviewers. Thanks to the efforts of our reviewers in 2022, the median time to first decision was 22 days and the median time to publication was 40 days. Regardless of whether the articles they examined were ultimately published, the editors would like to express their appreciation and thank the following reviewers for the time and dedication that they have shown *Medicina*:
A. D. MarinisKristina Lah TomulićA. Karin Navarro-MtzKrisztián GáspárAare MartsonKrzysztof NowosielskiAbbas Ali GaeiniKrzysztof ZielinskiAbbas BalouchiKshama GuptaAbbas BitarKuan-Han LeeAbdallah AlmaghrabyKui ZhangAbdolhassan KazemiKuldeep DoleAbdolreza JamilianKun WangAbdonas TamošiunasKun XuanAbdul GofirKun YangAbdul Razak MariatulqabtiahKun ZhengAbdul WaliKunal AjmeraAbdulkarim HasanKundan SivashanmuganAbdullah A. AlsabaaniKun-Hui ChenAbdullah DemirtaşKunihisa MiwaAbdullah GibrielKun-Yun YehAbdullah Md. SheikhKuofeng HungAbdulqadir J. NashwanKuotai ChenAbdulrahman AlsultanKurien Biji TheyilamannilAbhay K. PandeyKwang-Sup SongAbhijit Sukumaran NairKyle LamAbhiram KannegantiKyoung Sik ParkAbhishek KatakwarKyu Sung ChungAbraham Escobedo-MoratillaKyu Young ChoiAbraham Puga-OlguínKyung Eun LeeAcanfora DomenicoKyung Rae KoAchille MarinoLai Chih-ChengAchilleas BoutsiadisLakshmi MenonAdam HalinskiLan WangAdam KernLana NežićÁdám SchléglLanfranco D'EliaAdam TarnokiLars KästnerAdam WalkowiakLars P. KamolzAdebayo AdeyinkaLaura A. OrtmannAdela GoleaLaura BurattiAdelaida AvinoLaura CominiAdele WeinerLaura CortiAdiele DubeLaura FoxAdina FésüsLaura MaccaAdina Turcu-StiolicaLaura MarchiAditi KulkarniLaura MitreaAdli AliLaura Muñoz-BermejoAdolfo Francesco AttiliLaura PatrussiAdrian Vasile MuresanLaura SarnoAdriana G. Nevarez-FloresLaurence HamardAdriana GrigorașLaurent MachetAdriano FriganovicLaurent SuppanAdrie VerhoevenLaurent WillemotAdrija HajraLauri SoinneAfrouz AbbaspourLavinia DuicăAfshan BeyLayth Mula-HussainAfzal MisraniLazar VelickiAgam BansalLazaros TzelvesAgarwal TarunLech B. DobrzańskiAgata GabryelskaLei HeAgata JanowskaLei WanAgata MichalakLei ZhangAgata Skrzat-KlapaczyńskaLeigh KotzeAgata Sobczyńska-MaleforLeila AllahqoliAgathe BourgmayerLekshmi R. NathAgnieszka A. KołodzińskaLenka ŠpinarováAgnieszka Ćwirlej SozańskaLeon SeifertAgnieszka KordekLeonardo BascoAgnieszka KotalczykLeonardo CellenoAgnieszka KrólickaLeopoldo ArdilesAgnieszka LasotaLeopoldo Cosme-SilvaAgnieszka MatuszewskaLeping LiAgnieszka MlynarskaLesley CharlesAgnieszka Owczarczyk-SaczonekLeslie Marisol Lugo GavidiaAgnieszka SiennickaLevent ÖzçakarAgro Felice EugenioLia GinaldiAgustín Aibar-AlmazánLiana PlesAgustín Díaz-ÁlvarezLi-Ang LeeAgustín R. MirandaLiang WangAhila Singaravel ChidambaranathanLiat ChaushuAhmad Abdel Hamid ElheenyLiborija Lugovic-MihicAhmad GharaibehLiborija Lugović-MihićAhmadian ElhamLi-Ching ChangAhmar RaufLidija TodorovicAhmed A.H. AbdellatifLihong HaoAhmed Abdelbaset-IsmailLikai HuangAhmed AbdouLiliana A. Fidalgo-DomingosAhmed Abu-AwwadLiliana PorojanAhmed Abu-ZaidLin SongAhmed Adel AoudeLina T. Al KuryAhmed ElamragyLina YoussefAhmed ElmassryLinda JohanssonAhmed HusseinLinda PetersAhmed Yaseen AlqutaibiLinda TognettiAhsan Mohamed JawedLindawati S. KusdhanyAhsan Zil-E-AliLine RodeAida MetoLing LiAida PetcaLing TangAikaterini MantakaLinlin GuoAi-Ru HsiehLipin JiAishwarya GurumurthyLisa M. JenkinsAjay RajaramLisse Angarita DavilaAjitanuj RattanLital Keinan-BokerAjoy Prasad ShettyLi-Tu ZhangAkhilesh ShakyaLiu LeiAkihiko HiyamaLivio VitielloAkihiro KanamoriLiviu MacoveiAkilavalli NarasimhanLongbo ZhangAkinari SekineLorenzo AngeliniAkio OishiLorenzo CostaAkira IwataLorenzo FerriAkira MatsumoriLorenzo LippiAkira UmemuraLoreta KubilieneAkitoshi SanoLourenço Sbragia Neto SbragiaAlaa DaudLovorka BrajkovićAlaimo AlessandroLu LiAlana CampbellLu WangAlassane SeydouĽubica ĎudákováAlba CorellLuca AmbrosioAlban Fouasson-ChaillouxLuca CimaAlbert FujakLuca CostanzoAlbert RubbenLuca EspositoAlbert Thomas AnastasioLuca FerrettoAlberto BarbieriLuca GazziniAlberto De BiaseLuca GiannellaAlberto Di NapoliLuca MezzettoAlberto García-BarriosLuca OngaroAlberto GrassiniLuca Oscar Redaelli De ZinisAlberto LorenzattiLuca Paolo WeltertAlberto MavilioLuca ProsperiniAlberto MellaLuca RevelliAlberto Ofenhejm GotfrydLuca RicciardiAlberto RecchioniLuca Salvatore De SantoAlcibíades J. RodríguezLuca Salvatore MengaAlcy R. TorresLuca TagliaferriAldo Bruno GiannìLucia Costa-PaivaAlejandro Herreros-PomaresLucia GozzoAlejandro Rodríguez-VázquezLucia MalaguarneraAleksandar RakićLucia StančiakováAleksander KaniaLucio Della GuardiaAleksander Rygh HoltenLucretia UdrescuAleksander ŚlusarczykLucrezia BottalicoAleksandr A. RubelLucy NorrisAleksandr PoliakovLuigi CerboneAleksandra D. DoronjskiLuigi Di FilippoAleksandra Gaworska-KrzemińskaLuigi IraceAleksandra JoticLuigi LoscoAleksandra Karczmarska-WódzkaLuigi MeccarielloAleksandra KołotaLuigi RuggieroAleksandra KrólikowskaLuigi VenturaAleksandra S. KristoLuigi VivonaAleksandra StupakLuis FernandezAleksandra SzylińskaLuis FonteAleksandra SzymczakLuis Rafael Moscote-SalazarAleksandra WardzyńskaLuis RodrigoAleksandras VilionskisLuisa BarrosAlen DžuburLuis-Alfonso Arráez-AybarAleš BlincLuiz Fernando Romanholo FerreiraAlessandra AldieriLuiz Vinicius De Alcantara SousaAlessandra FilosaLuka FlegarAlessandra LaforgiaLukas RasulićAlessandro AntonelliLukasz BuldakAlessandro CeliŁukasz JanczewskiAlessandro CuomoŁukasz KlepackiAlessandro De SireŁukasz KruszynaAlessandro FeolaLukasz MalekAlessandro GalaniŁukasz MałekAlessandro GranitoŁukasz PałkaAlessandro MalobertiŁukasz RydzikAlessandro PosaLuminiţa Maria NicaAlessandro RamieriLung-Chi LeeAlessandro RizzoLynda R. CorriganAlessandro TelLyubov E. SalnikovaAlessandro TonacciM. Dulce EstêvãoAlessandro VittoriM. Rezaul IslamAlessia PaganelliM.S. MuthuAlessio ContiMª Lourdes Del Río-SoláAlex AlfieriMaciej DyrbuśAlex JosephMaciej J. KotowskiAlex NiuMaciej JedlińskiAlex Siu Wing ChanMaciej JozwikAlexander A. PugovkinMaciej MichalikAlexander B. MohsenyMaciej StaporAlexander BerezinMaciej W. SochaAlexander Dimitrov ShinkovMacrina B. Silva-CázaresAlexander GorokhovskyMadalina Irina MitranAlexander Maximilian EickhoffMaddipatla ReddikumarAlexander NivenMadhan JeyaramanAlexander V. OleskinMadhavan NampoothiriAlexandra GratlMadhuradhar ChegondiAlexandra LazarMagda ZanelliAlexandra MateiMagdalena Knetki-WróblewskaAlexandra PotekhinaMagdalena KostkiewiczAlexandra Vink-NieseMagdalena Mackiewicz-MilewskaAlexandre Gonzalez-RodriguezMagdalena WójciakAlexandre González-RodríguezMagdalena ŻychowskaAlexandrina MunteanMaher RashwanAlexandrina S. NikovaMaheswari MuruganandamAlexandros DaponteMahmoud ShehataAlexandros LaiosMahmoud SmidaAlexandru Bogdan CiubaraMahmoudreza HadjighassemAlexandru BucurMahshid NaghashpourAlexandru BurlacuMainul HaqueAlexandru CorlateanuMairi PucciAlexandru Florin RogobeteMaite E. Rivera GorrínAlexandru UliciMaja L.J. ŽivkovićAlexey G. KruglovMaja MiletićAlexey SarapultsevMajed A. RefaaiAlexey SuminMajed AlamriAlfonso TortorellaMajed H. WakidAlfredo Cruz-GregorioMajid KoozehchianAlfredo G. CasanovaMakoto HaradaAlfredo Merino-LunaMakoto TakeiAlfredo Parra LucaresMałgorzata KlimekAlgimantas TamelisMalgorzata KolpaAli Abbas RizviMałgorzata PasekAli Al KaissiMałgorzata PrzybyłoAli Kerim YılmazMalgorzata Sokolowska-WojdyloAli SaeedMałgorzata Zalewska-AdamiecAlice Chiara ManettiMalik AydinAlice Mirela MurariuManan ShahAlina Daciana ElecManazir AtharAlina WilkowskaManfred RauhAlireza NazarianMangesh HadeAlisa JohnsonMangesh Prashant JoshiAlisha DhimanManish CharanAlison GrazioliManish JainAlkhateeb AbedalrhmanManlio SantilliAlla KushnirManoj Kumar TembhreAlok KumarManoj ThapaAlok SudManuel Durán-PovedaAlpamys IssanovManuel Frías VargasAlper SozutekManuel GranellAlpo VuorioManuel Marques FerreiraAltaf KondkarManuel Montero-Pérez-BarqueroAlvarado-Ibarra MarthaManuel MuroAlvaro DowlingManuel StruckÁlvaro Zubizarreta-MachoManuel WilbringAmadeus DobrescuManuela LenghelAmanda V. SardeliManuela MeirelesAmany MohamedMao XumingAmany SholkamyMara CarsoteAmar ParvateMarai IbrahimAmbar Lopez-MacayMarc DeanAmbreen AzharMarc HanschenAmbrina A. QureshiMarcel AdlerAmbuga BadariMarcel NaikAmeesh M. IsathMarcello CandelliAmelia PietropaoloMarcello Della CorteAmir HadiMarcello RotaAmir Reza RoknMarcelo Haertel MiglioranzaAmirhossein RahavianMarchel S. VetrileAmirmasoud AhmadiMarcia ConventoAmit K. MaitiMarcin DerwichAmit PalMarcin GierekAmjad Islam AqibMarcin KosmalskiAmolak BansalMarcin KurzynaAmrita PaiMarcin OpławskiAmy D. DobberfuhlMarcin SzerszeńAmy L. ShaverMarco CaricatoAna Angelova VolponiMarco CascellaAna BertoniMarco CasciaroAna BoroveckiMarco ChiostriAna Carla Raphaelli Nahás-ScocateMarco DolciAna Catarina Pronto-LaborinhoMarco FoganteAna CusumanoMarco La VerdeAna DascaluMarco MarcascianoAna HladnikMarco MascittiAna Leticia Fornari CapraraMarco MaterazzoAna Luiza De Carvalho FelippiniMarco PaciottiAna Maria CiubarǎMarco PalumboAna RamosMarco PassiatoreAna RochaMarco PicardiAna Sofia CostaMarco SeracchianiAna Sofia OliveiraMarco TorresAna Teresa Corte-RealMarco ZuinAna Vujaklija BrajkovićMarc-Olivier GauciAnaïs TuepkerMarcos BrioschiAnamaria CristinaMarcus LancéAna-Maria OproiuMarek BolanowskiAnand MaryaMarek CzosnykaAnandharajan RathinasabapathyMarek DurlikAnant JainMarek MalikAnanto AlhasyimiMarek NahajowskiAnastasia BougeaMargarida Freitas-SilvaAnastasia N. SergeevaMargherita EufemiAnbukkarasi MuniyandiMarguerite R. DuaneAnca BobircaMaría Angustias Sánchez OjedaAnca BojanMaria Anna MessinaAnca SimionescuMaría Antonia Parra-RizoAndi Masyitha IrwanMaria Blanco-DiazAndras BanvolgyiMaría C. Jiménez-MartínezAndras BikovMaria C.T. CangussuAndraz DovnikMaria Carmela Di RosaAndré CorreiaMaria Carolina MendesAndré Lopes SaraivaMaria Chiara GattoAndre Luiz CostaMaria ContaldoAndre RodackiMaria CotelliAndre WirriesMaria Cristina MondardiniAndré WirriesMaría De La Concepción Noriega MatanzaAndrea AbateMaría Del Carmen García-RíosAndrea BrambillaMaria Del Mar Pacheco HerreroAndrea BrugnoloMaría Dolores Marrodán SerranoAndrea ButeraMaría Eulàlia Fernández-MontolíAndrea CassoniMaria Fátima PaulinoAndrea CortegianiMaría Fernanda Bernal-OrozcoANDREA De VitoMaria FerraraAndrea Duarte DoetzerMariá Garciá-MonteroAndrea FaggianoMaria GoulielmakiAndrea GianniniMaria HeinrichAndrea L. HildebrandMaria Helena Melo LimaAndrea LaurenziMaria I. ZervouAndrea Luísa Fernandes AfonsoMaria IbrahimAndrea MichelerioMaría José Bautista-LlamasAndrea Milostić SrbMaría Julia MartínAndrea MozzarelliMaria KouroupiAndrea SaladinoMariá Librada Porriño-BustamanteAndrea SambriMaria Luigia RandiAndrea SistiMaria Maddalena MarrapodiAndrea SpecialeMaria MatveevaAndrea TarozziMaria NetoANDREA TINELLIMaría Orosia Lucha-LópezAndreas BusjahnMaria P. NtaloukaAndreas KoulourisMaria Paola BertuccioAndreas LipphausMaria Rayane Correia De OliveiraAndreas S. PetersMaría Reina BuenoAndreas StadlerMaria SarapultsevaAndreas Wolfgang ReskeMaría SerramitoAndreas ZielkeMaria SimonenkoAndreea BorleaMaria TsiarliAndrei HavasiMaria V. CorriasAndrei Marian FeierMaria Vaz-PattoAndreia QueirozMaría Yolanda Castellote-CaballeroAndrej MarkotaMaria Zadarko-DomaradzkaAndrés E. QuesadaMaría-Carmen Sánchez GonzálezAndres Reinoso-CoboMariachiara CarestiaAndreu Comas-GarciaMariagiovanna CantoneAndrew ChanMaría-Jesús Tabernero-DuqueAndrew Guy MessengerMariana BenderskyAndrew K. GormleyMariana Fragão-MarquesAndrew McCombieMariana SantosAndrey Jorge SerraMariann HarangiAndrey KechinMarianna K. TheodoraAndrijana VčevaMarianna KapsetakiAndrzej BiedermanMariano MascarenhasAndrzej KomorowskiMarie Agnès BringerAndrzej KutarskiMarie Hélène ErreraAndrzej TomczakMarie Saghaeian JaziAndy Wai Kan YeungMarie-Agnes DocquierAndy Wei-Ge ChenMariia NesterkinaAnestis KarakatsanisMarija HabijanAneta WieczorekMarija M. BožićAnette KjeldsenMarija RoguljićAngel León-BuitimeaMarija VukojaÁngel SevillanoMarijana CurcicAngel Vértiz-HernándezMarilena GregoriniÁngela Ayén-RodriguezMarilyn AderAngela Quispe-SalcedoMarimar M. Saez De Ocariz-GutierrezAngeline Hui Cheng LeeMarina Lapter-VargaAngelo Alessandro MarraMarina Mat BakiAngelo CarrettaMarina MichalakiAngelo RuggieroMarina MuzzaAngelo TorrenteMarina Ruxandra OteleaAniello MaieseMarino ParoliAnil TailorMarino SignorelliAnil TharappelMarinos KosmopoulosAnis CerovacMario César Salinas CarmonaAnissa ZaouakMario CoronaAnjana BairagiMario CostantiniAnn Christin PecherMario DiplomaticoAnna AureliMario Enrico CanonicoAnna BrzeckaMario LaganovićAnna CampanatiMario Mendoza-SagaonAnna DanielewiczMărioara ȘimonAnna EsparhamMarios RossidesAnna Kasielska-TrojanMarissa J. CarterAnna Kerpel-FroniusMarius BoariuAnna ManolopoulosMarius CrainaAnna Maria SchitoMariusz Aleksander BromkeAnna Michalak-StomaMariusz SikoraAnna Olasińska-WiśniewskaMariya LivzanAnna Paradowska-StolarzMarjan SabbaghianAnna PietrzakMark J. LambrechtsAnna PodlasekMarko BaškovićAnna RzepakowskaMarko KumrićAnna StaniszewskaMarkus GessleinAnna StarzyńskaMarkus VeltenAnna ViolaMarlina LovettAnna Zalewska-JanowskaMaroš HrubinaAnnalisa PaceMarta Blasco-AlonsoAnnamária ErdeiMarta FijałkowskaAnnette B. PfahlbergMarta NardiniAnnie Lee See MunMarta OsrodekAnnunziata NuscaMarta Rodríguez-HernándezAnthony L. KovacMarta SarkozyAnthony ValverdeMarta Waliszewska-ProsółAntimo CutoneMarta Z. PaciaAntoine BioyMartín A. MaraschioAntoine NaemMartin BeerAnton MoritzMartin Bjørn StausholmAntonella ArgentieroMartin HartrumpfAntonella Di SottoMartin KapitánAntonella TufanoMartin KopaniAntoni FellasMartin LovečekAntonia KondouMartin M. MortazaviAntonija TadinMartin RakusaAntonio Bayes De LunaMartin Schmidt-HieberAntonio Córdoba FernándezMartin TeraaAntonio De LucaMartina SmolićAntonio Giovanni SolimandoMartínez-Ramón Juan PedroAntonio GloriaMarwa A.A. FayedAntonio GuaitaMarwa GaberAntonio NennaMarwa MatboliAntónio QueirósMarwan El MobadderAntonio RomanelliMary M. SchneiderAntonio Simone LaganàMaryam SalimiAntonio SteccoMarzia SegùAntonio Victor Campos CoelhoMasafumi InomataAntony Herold PrabhuMasaharu HataAnudeepa SharmaMasahiko TsuchiyaAnudishi TyagiMasahiro TakahashiAnuli NjokuMasahito KatsukiAnurag SatpathyMasaki OkamotoApichat KaewdechMasakiyo YatomiApostolos AgrafiotisMasanori MurakamiApostolos D. GalatosMasanori TeshimaApostolos Georgiou PappasMasfueh RazaliApostolos I. TsolakisMasoud KeikhaApostolos MamopoulosMassimiliano AmmirabileAqel AlbuttiMassimiliano BianchiArbanasi EmilMassimiliano De PaolisArbaz SajjadMASSIMILIANO MANCINIArgyro MaziotiMassimo CapocciaArif SiddiquiMassimo CarossaAriful HaqueMassoni FrancescoArisan VolkanMasutaka FurueArjan VissinkMatas JakubauskasArkady KotlyarMatei BratuArmando Silva-AlmodóvarMatej StrnadArminas JasionisMatej StuhecArnaud AlvesMateja SiršeArne KienzleMateusz BabickiArnulfo Ramos-JiménezMateusz JankowskiArshi KhanamMateusz SpiewakArtem OvchinnikovMatevž TomaževičArthur De Sá FerreiraMatheus BertanhaArtur GądekMathias TrempArtur PasternakMathilde SengoelgeArturo SantosMatias IglickiArul ChandranMatija BarbičArulselvi SubramanianMatteo BolcatoArun KhattriMatteo CarpiArun MeyyazhaganMatteo MatteucciArun PrasathMatteo MonticelliArun RajasekaranMatteo MorottiArun-Kumar Kaliya-PerumalMatteo NardinAsaf AchironMatteo RenzulliAshish JainMatteo RipaAshok KumarMatthew BottomleyAshuin Kammar-GarcíaMatthew DevallAsif HameedMatthew J. KesterkeAsif KhaliqMatthew J. RegulskiAsif NawazMatthias D. HoferAsma Ismail MahmodMatthias J. RoggendorfAssunta PozzuoliMatthias JentschkeAstrid L. GiraldoMatthias KarlAtari MaherMatthias KorellAthanasia ProklouMatthias SteinertAthanasios PapadopoulosMatti A. PenttiläAthanasios TragiannidisMattia BarbotAthikhun SuwannakhanMattia DominoniAthina A. SamaraMatúš DohálAtiyeh M. AbdallahMaureen HansonAtsushi FujioMaurice MarsAtsushi TeramotoMauro CervigniAttila JakabMauro GiacomelliAttila OláhMaxwell SuAudrey Opoku-AcheampongMayur VirarkarAudrius DulskasMazapuspavina Md-YasinAurel PeraMazza MariannaAureliusz KolonkoMd MohiuddinAušra Lukošiūtė-UrbonienėMd Rabiul IslamAušra MongirdienėMebin MathewAvinash KumarMeer M. ChisthiAwadhesh AryaMeerambika MishraAxel SteigerMegha SharmaAya SugiyamaMeghal GagraniAyataka FujimotoMehdi AlikhaniAyelet Zlotogorski-HurvitzMEHMET ALPHAN ÇAKIROĞLUAyman ElsamanoudyMehmet BaysalAyman MahmoudMehmet GülAyse AyhanMehmet Sühha BostanciAyyanar SivananthamMehrez ElnaggarAzalia Avila-NavaMehrnoosh TashakoriAzzawi HadiMelania BalasBabett RamsauerMelissa Anne SuterBadiah BaharinMelita Vukšić PolićBahaa NasrMeng WangBaharudin AbdullahMengxi ShenBaki HekimoğluMériam SabbahBakri M. AssasMeritxell MunmanyBala S. C. KoritalaMerja KallioBalakrishna KoneruMete IşıkoğluBalázs CsomaMichael CampbellBalázs MargitMichael CottonBalestra CostantinoMichael EfremidisBálint TamaskovicsMichael EwerBaljinder Singh DhinsaMichael Ke WangBaltatu SimonaMichael L. PretterklieberBaofeng WangMichael LentzeBarani Kumar RajendranMichael LoughlinBarbara AntunesMichael McCroryBarbara BrognaMichael NorbergBarbara Di StefanoMichael PretterklieberBarbara FuenzalidaMichael SchwakeBarbara Mara KlinkhammerMichael T. KoltzBarbara TomasinoMichail PapapanouBárbara Vieira Do LagoMichał BorysBarbosa Daniel JoséMichał GontarzBarisic KarmelaMichal J. DabrowskiBarry SwerdlowMichał Kazimierz ChojnickiBart De GeestMichał KosowskiBart PijlsMichał MączewskiBartłomiej GmajMichał MichalakBartosz MałkiewiczMichał PlutaBasel AbdelazeemMichal SzeremetaBassant BarakatMichał-Goran StanišićBayan AlsaidMichalis GalanopoulosBayu Satria WiratamaMichel R. PopoffBeata Jankowska-PolanskaMichela RossiBeata NowakMichele BoffanoBeata Świeczko-ŻurekMichele CorrealeBeatrice CairoMichele Di CosolaBeatrice LeonardiMichele FioreBeatriz Navarro-BrazálezMichele GuidaBebiana CondeMichele PaciottiBee K. TanMichele PietragallaBegoña Polonio-LópezMichele Schiano Di VisconteBegüm OkutanMichele ValmasoniBéla FaludiMickael EssoumaBen Abdallah IsmailMicol MassimianiBen MulhearnMiguel Angel RubioBenedetta NacmiasMiguel R. Pecci-LloretBenedict FrancisMiguel RicouBeng Fye LauMiguel Urina-TrianaBeniamin Oskar GrabarekMihaela Flavia AvramBenjamim FicialMihaela GhitaBenjamin Barsties Von LatoszekMihaela HedeșiuBerceste GülerMihaela HostiucBerenice Caneiro-QueijaMihaela MocanBerger ZoltánMihaela MoscaluBergita GanseMihaela PerteaBernhard N. BohnertMihai CapilnaBernhard RauchMihai CenariuBert VandenberkMihai DimitriuBertha Alejandra Martinez-CannonMihai Stefan MuresanBettina BlaumeiserMika NonoyamaBeyza GoncuMikk JürissonBharani Krishna Mynampati ArunadithyaMi-Kyoung ChoBhaskar DasMi-Kyung KimBhavisha BakraniaMilan RadovanovicBi Ning ZhangMilena G. MilutinovićBiagio BaroneMilena ŚwitońskaBiagio RaponeMilena VasicBianca Laura CinicolaMilica DeklevaBianca Lopes Cavalcante-LeãoMilica NedeljkovićBibhusal ThapaMiljana JovandaricBiju SomanMiloš BjelovićBindey KumarMiloš StankovićBinu Kandathilparambil SasiMina HurBirkan BozkurtMinerva Gomez-FloreBirol TopçuMing ChenBirute ZablockieneMingchung ChouBiswadeep DasMing-Jen TsaiBjörn SchreiweisMing-Jui HungBlas LeandroMingjun LiuBo WangMing-Lung ChuangBo ZhaoMingming TongBo-Chul ShinMing-Yan JiangBogdan CulicMingyi ChenBogdan DorofteiMing-Yi ShenBogdan Ionel TambaMin-Ho HanBogdan ObrzutMin-Hsien ChiangBogdan PavelMinjeong NamBogdan Silviu UngureanuMinyahil WolduBogdan SoceaMiodrag JanicBogdan VeliceasaMiran ŠebeštjenBojan JorgićMiranda Miranda Muhvic-UrekBojan KrebsMiranda Muhvic UrekBojan MasanovicMircea-Catalin FortofoiuBolaji Lilian Ilesanmi-OyelereMirela TiglisBoohwi HongMiren García-CortésBoon-Huan TanMiriam HickeyBorek SehnalMirjana StojkovićBoris Filipović-GrčićMirna A. FawazBoris KozlovMiro JukićBoris MraovicMiroslav HercegBorkowska AlinaMiroslav VaverkaBortolo MartiniMiroslav VujasinovićBo-Young HongMiroslaw WielgosBożena SobkowiczMiroslawa PuskullogluBożena Targońska-StępniakMirthe H. SchootsBrandon Peter Lucke-WoldMirto FolettoBranislav KollarMiruna NemeczBranislava TeofilovicMirza PojskicBrega CarlottaMirza PojskićBrett HamblyMisao NishikawaBrian GreeleyMisbahuddin M. RafeeqBridget M. BarkerMislav MikusBrijeshkumar PatelMitja RuprehtBritt OpdebeeckMitra AkbariBroglie Martina AnjaMitsushige SugimotoBruno ChrcanovicMi-Young LeeBruno FiondaMobashir MohammadBruno TrovatoMoema HausenBumbaširević UrošMohamed A. AbdallahByung-Kwan SeoMohamed A. HamidCácia SignoriMohamed Abdullah JaberCagetti Maria GraziaMohamed AonÇağrı ErginMohamed FarghaliCaitlin MitchellMohamed Salih MahfouzCaitlin TakahashiMohammad AkbariCaizer CosticaMohammad Al-WardatCălin Bogdan ChibeleanMohammad EtoomCalixto MachadoMohammad KamranCamelia CoadaMohammad KhalilCamila Squarzoni DaleMohammad Reza AminiCarlo BizMohammad Shamsul OlaCarlo DoriaMohammad Yasir EssarCarlo PerisanoMohammadreza ShalbafanCarlo RonsiniMohammed DalliCarlos Alberto CañasMohammed GrawishCarlos Curvelo SantanaMohammed NadarCarlos Díaz-CastilloMohammed NassarCarlos DurántezMohammed SayedCarlos E. VelascoMohammed ZayedCarlos Enrique Cuevas SuárezMohan Kumar KalaiahCarlos GasparMohd Abul KalamCarlos Hernando GómezMohd FarhanCárlos L. Ayán PérezMohd ImranCarlos López-de-CelisMohd Yusmiaidil Putera Mohd YusofCarlos Martin LlorenteMohit JoshiCarlos Miguel MartoMohit KakarCarlos OrozcoMohit Kumar PatralekhCarlos Rocha-De-LossadaMohmed Isaqali KarobariCarlos SalaveraMohsen KerkeniCarlos UbedaMohsin KhurshidCarlos VasconcelosMohsin MaqboolCarlton DampierMoises Alejandro Alatorre-JimenezCarmen NeamtuMoises Armides Franco-MolinaCarmen Nicoleta PurdelMoises DiagoCarmen PanaitescuMoisés MarquinaCarmine ConteMoito IijimaCarmine PiconeMojdeh BanaeiCarolina RojasMojtaba AnsariCarolina TorresMolly YaoCaroline M. SpeksnijderMominur RahmanCaroline SubraMona BoazCarolyne FalankMona ZvancaCaseiro ArmandoMonica GallegoCassandra E. HolbertMonica Livia GheorghiuCatalin TiliscanMónica Ríos-SilvaCaterina BuffoneMonika KoziełCatherine BlanchetMonika KujdowiczCátia DominguesMonika Łopuszańska-DawidCecilia Alegado JimenoMonika Michalek-ZrabkowskaCecilia BindaMonika PrakashCecilia BoveMonika SobocanCecilia Li-TsangMonlezun Dominique JCecilia Maria VeraarMontalti RobertoCecilia NalliMordechai PollakCecilia XimenezMorgan D. SalmonČelakovská JarmilaMoritake IguchiCera NicolettaMoritz MarkelCesar Lopez-CamarilloMorteza Fallah KarkanChan Jason YongshengMostafa El KhatibChan ParkMotaz HamedChandramani PathakMotofumi SuzukiChang Chiung-ChihMuhamed AminChangwei WeiMuhammad Adeel AhmedChangwon JeongMuhammad AfzaalChan-Ho ParkMuhammad AsimChan-Young KwonMuhammad Hammad ButtChao LeiMuhammad ImranChao YangMuhammad IqhrammullahChaochao ZhouMuhammad KhanChao-Hsien ChenMuhammad N. ZahidChao-Yie YangMuhammad NoumanChappard ChristineMuhammad ShahidChariklia Tziraki-SegalMuhammad Sohail KhanCharles AllenMuhammad ZafarCharles William YoxallMuhammed Sedat SakatCharlotte SteenblockMuhanad S. AlharekyCharu Mohan MaryaMukherjee AnupamChaves-Martinez Felipe JavierMukhtar AnsariChawla KiranMumtaz AnwarChen Guo KerMun-Ho RyuChen ZhuMuralikrishna LellaCheng-Chieh YenMurat AladaǧCheng-Hsu WangMurty ManthaChen-Long WuMushriq AbidChenmala KarthikaMustafa AkkayaChen-Wang ChangMustafa ÖztürkCheol Hee ParkMustafa TascanovChia-Hung ChouMuthu SathishChiaki SatoMya Myat Ngwe TunChiao-Nan ChenNabil NicolasChiara FoglieniNada SallamChiara PolichettiNadia NajafiChiara SorberaNadica MiljkovićChiarella GiuseppeNadine S. AguileraChibueze AnosikeNadinne RomanChien Chih WangNadira Y. YuldashevaChien-Chang KaoNaemeh NikvarzChiencheng ChenNafisa JadavjiChien-Chih ChenNaga Babu ChinnamChienhsiu HuangNagehan AslanChien-Jen HsuNahir MenachemChih-hao ChangNaiara OzamizChih-Hao ChenNai-Jen ChangChih-Hsin HsuNajm AlshahraniChikako HaraNan JiangChina NaganoNanae TanemuraChing Ju ShenNaohiro ShiojiChing-Chung HsiaoNaoto NagataChing-Yi ChangNarayan RamamurthyChi-Nien ChenNarcis Octavian ZãrnescuChinyerum OpuwariNardos AbebeChisci GlaucoNaresh DamukaChi-Wei LinNaresh KojuChizu SanjobaNareswari CinintaChloe Miu MakNarimantas Evaldas SamalaviciusChongyang DuanNark-Kyoung RhoChoo Hock TanNaseer AhmedChris H.L. LimNatalia DłużniewskaChris H.L. LimNatalia DowgiałłoChrist MichaelNatalia GeppeChristian BleilevensNatalia MotasChristian Di FlorioNatalia V. BeloborodovaChristian Von RüdenNatalia V. GulyaevaChristiane GerardNatalia Viktorovna NizyaevaChristina BothouNatalie EnninghorstChristine AustinNatalija VedmedovskaChristine FardeauNatalya A. ShnayderChristine StierNatarajan AyithanChristophe BoulayNataša Marčun VardaChristophe PrehaudNataša RančićChristopher J. YoungNathalia Carolina Fernandes FagundesChristopher M. MaulucciNathalia PadillaChristos K. YiannakopoulosNathalie Grace ChuaChristos KroupisNathaniel MercaldoChrysoula PerdikogianniNattawat KlomjitChun Hung ChuNauman Rahim KhanChung-Cheng WangNebojša D. ZdravkovićChung-Chien HuangNebojša JasnićChun-Gu ChengNEBU ABRAHAM GEORGEChun-Hsien YuNeda HakimihaChunping YuNeerja BhardwajChun-Ting LiNeil GlossopChun-Wu TungNémeth BalázsChyntia JasirwanNenad PandakCiavoi GabrielaNesrine Salah Dine El-SayedCiesla MaciejNéstor Ventura-AbreuCiro ImbimboNeubauer HansCirulli NunzioNeusa Yuriko Sakai ValenteClaps FrancescoNevin KocamanClaudia Angela Di SilvestriNewman OsafoClaudia KedorNgozi Immaculata UgwuClaudia Reyes-GoyaNguyen Minh DucClaudia TodaroNicholas A. GiovincoClaudia Tovar-PalacioNicholas GrubicClaudiane FukuchiNicholas J. AndersenClaudio BernardazziNicholas ReedClaudio FeoNick VordosClaudio VelatiNicola MadugnoClaudiu MargineanNicola MarottaClaudiu N. LunguNicola MondanelliClaus MosekeNicola MontemurroClemax Sant'AnnaNicola TroisiClément BarsNicolae GicaClemente Maria IodiceNicolae PopaClifton HolmesNicolas GendronConcetta Di NoraNicoleta AntonConstantina AggeliNicolina CapoluongoCorentin PangaudNidhi ManaktalaCorina Marilena CristacheNiels Damkjær OlesenCorina VasileNikhil N. VermaCornel MihalacheNikhil Suresh BhandarkarCornelius EwuosoNiklas PakkasjarviCorrado CiattiNikol SulloCosmeri RizzatoNikola StojanovicCosmin FaurNikolai PaceCosmin PuiaNikolai RamadanovCraig PearlNikolaos A. ChrysanthakopoulosCraig R. BaileyNikolaos AntonakopoulosCrescenzio SciosciaNikolaos E. TzanakisCrischentian BrinzaNikolaus BörnerCristi TartaNikolaus HaasCristian FurauNikolay BugaevCristian IndinoNikos ViazisCristian MirvaldNilakshi BaruaCristian PersuNimisha Chinmay ShahCristian ScheauNina KrügerCristian TrambitasNinghai GanCristiana Iulia DumitrescuNirmal Kumar MohakudCristiano AmarelliNiroj Kumar SahooCristiano CarbonelliNishant Ganesh KumarCristiano De FrancoNitasha PhatakCristian-Radu JecanNitin BansalCristina Andreea AdamNitin KhandelwalCristina B. NevesNitin KumarCristina BicaNitinkumar BorkarCristina CapatinaNizamettin GüzelCristina CortisNobuaki MichihataCristina M. JorgeNobumichi KobayashiCristina MambetNoemí Cárdenas-RodríguezCristina MazzaccaraNoemi SalmeriCristina MondelloNoha Mousaad ElemamCrystell Guadalupe Guzmán PriegoNoordam CeesCuilan LiNor Norazlin KamalCyrus MotamedNor Rafeah TumianDace RezebergaNorbert GrafDagmar PavluNorbert KissDaiji NagayamaNoreen J. HickokDaisuke ObinataNorihiro IsogaiDaisuke SakaiNorio ImaiDale QuestNorio YamamotoDalia ZaliunieneNorizzati AmsahDalila ScaturroNorma Velázquez-GuadarramaDamiana ScuteriNouran Yousef SalahDamir GrebićNowicki Grzegorz JózefDana LawrenceNuman DedeoǧluDana Liana StoianNuman MajeedDana Roxana BuzasNuno MoraisDandan WuNur Syamimi AriffinDane KrtinicNutjaree JeenduangDaniel Andrei IordanNuwadatta SubediDaniel BiermannNydia Tejeda MunozDaniel BléroNzwalo HipolitoDaniel Garcia-OvejeroOana AlmasanDaniel GrassoOctav Marius RussuDaniel Griffiths-KingOctavian AndronicDaniel JanitschkeOctavian EnciuDaniel Landero HuertaOğuzhan MeteDaniel LighezanOhsawa SuguruDaniel ParamythiotisØistein HovdeDániel PécsiOkuno MitsuruDaniel PintoOla KlevelandDaniel Robert SchlattererOlav HaugenDaniel ŚlęzakOlga C. RojasDaniel VeghOlga GordeevaDaniel WendtOlga GutkowskaDaniel WoldringOlga MalyshevaDaniela BuchaimOlinic Dan MirceaDaniela ChlíbkováOliver BischelDaniela DamianiOliver Heinrich FelthausDaniela MiricescuOliver SanderDaniela Opris-BelinskiOliver VasiljDaniela PaňkováOlivera Mitrović AjtićDaniela PintoOm Prakash ChoudharyDaniela RossinOmar El MeligyDaniele LinardiOmar El ShahawyDaniele UrsoOmar Guzman-QuevedoDanijela TasicOmar KujanDanilo Marcelo Leite Do PradoOmeed NeghabatDario CalafioreOmer DzemaliDario LipariOmkara Swami MuddinetiDario MessenioOnur HapaDariusz ŁątkaOrawan KeeratisirojDariusz SitkiewiczOren PleniceanuDariusz SkabaOrietta SeguraDariusz TimlerOrouba AlmilajiDarko KeroOsamu KumanoDarko ModunOscar Figueras-AlvarezDarlene MitranoOskar RosiakDarren StoryOskars KalejsDavid DahlgrenOsmar Antonio Jaramillo-MoralesDavid GendelbergOsmindo R. Pires JúniorDavid Hernández-GuillénOsnat BashkinDavid José Casimiro AndradeOsvaldo MazzaDavid Mancha-TrigueroOthman Bin-AlamerDavid ManninoOthon PapadopoulosDavid Sancho-CantúsOumaima AmmarDavid TarnokiOurlad Alzeus TantengcoDavid ZweikerOzum ErkinDavide BavaroP. Anil KumarDavide BizzocaPablo Hernandez-LucasDavide BorroniPacini MatteoDavide De CiccoPai-Mei LinDavide RaineriPajor LászlóDawid StormanPalaniraja ThandapaniDa-Yong HouPalmina P. Petruzzode Asmundis CarloPalpu PushpangadanDe Martinis MassimoPanagiotis KorovessisDebdeep MitraPanagiotis KoulouvarisDebmalya BarhPanda SantoshDebora ColangeloPandora PoundDébora Do Rocio KlisiowiczPaola AntoniniDeborah A. LanniganPaola BonavolontàDeborah McCurdyPaola CiriacoDeepak KotiyaPaola MuggeoDeepak Kumar KhajuriaPaola QuaresimaDejan JakimovskiPaola SavoiaDelia HorhatPaola SenaDengfu YaoPaolo Albino FerrariDenis MiyashiroPaolo CompagnucciDeniz ÇankayaPaolo DorettoDennis FlanaganPaolo GroffDerek TranPaolo MarraDer-Shin KePaolo MartellettiDespina PantelidouPaolo RighiniDessy RachmawatiPaolo VincenziDevendra BansalParas N. YadavDevrim TarakçiParaskevi GoggolidouDhananjay YadavParaskevi TrivilouDhaval ChauhanParasvi S. PatelDhiran VergheseParente PaolaDhiya Al-JumeilyParham Jabbarzadeh KaboliDi Fiore AdolfoParis JafariDiana GiannarelliParvinder KumarDiana Marcela CastilloPasquale EspositoDiana Popa-AndreiPasuk M. MahakkanukrauhDiana ȚînțPathak Chandramani M.Didem OǧuzPatricia Acosta-VargasDiego DantasPatrícia Manarte-MonteiroDiego NogueiraPatricia Takako EndoDieter BettelheimPatrick G. BradyDillon MintoffPatrick Michael McNuttDimitri PoddighePatrizio TressoldiDimitrios KotsirisPatrycja KleczkowskaDimitrios MakrakisPatrycja Rzepka-WronaDimitrios PanagopoulosPatrycja S. MatusikDimitrios PatouliasPaul CummingDimitris MandalidisPaul I. HeidekruegerDimitris TatsisPaul Jonathan RochDimitris TsakogiannisPaul JülicherDimitris ZacharoulisPaula D. PrinceDina Di GiacomoPaula NelasDinesh RokayaPaulina AdamskaDineshani HettiarachchiPaulina KrawiecDionysios C. VorosPaulina MertowskaDivakar KaranthPaulina Szczepanik-KułakDivya BeriPaulo Goberlânio De Barros SilvaDivya GopinathPaulo J. PalmaDjordje PopovićPaulo PalmaDmitri ShchekochikhinPaulo SanchesDmitriy Nikolaevich AndreevPavel H. Lugo-FabresDmitriy VidermanPavel Ivanovich KrzhivitskiiDmitry S. PanfilovPavol SzomolanyiDoddy Moesbadianto SoebadiPaweł DomagałaDoerte WichmannPawel MiotlaDoina A. TodeaPaweł Petkow-DimitrowDomen PlutPaweł PiepioraDomenico DalessandriPaweł PiszkoDomenico FengaPaweł RamosDomenico GalassoPaweł WańkowiczDomenico Maurizio ToraldoPayam DibajDomenico PlantonePeđa KovačevićDomenico SolariPedro BullonDominic AugustinePedro FreireDominik DłuskiPedro Paulo Menezes ScariotDominik PromnyPei-Chen LiDominik PruskiPei-Wei ShuengDominika SkolmowskaPekka PorelaDominika ZającPenelope TrimpouDomiziano TarantinoPeng WuDöndü Üsküdar CansuPérez Plasencia CarlosDong Hyuk KangPetar AvramovskiDong LiuPetca Razvan-CosminDongfang LiPeter De Nully BrownDong-Man RyuPeter GoodwinDongmei LiPeter KeoghDongwei ZhangPeter Klein-WeigelDongxu ChenPéter L. KanizsaiD'Oria OttaviaPeter L. ToogoodDorin IonescuPeter MichielsenDorota NowosieleckaPéter Miklós KőmívesDorota OrtenburgerPeter MwangiDorota Pojda-WilczekPéter NémethDouglas P. BlackallPeter PaalDr. Sunayana Begum SyedPeter PikoDragan DjordjevicPeter RumneyDragana RadovanovićPeter. C. TaylorDragica SelakovicPetrick Ramesh A.L.K. PeriyasamyDragoș MarcuPetrisor Aurelia GeavleteDragoș PaladePetros IoannouDragos SerbanPeyman MirtaheriDrashya SharmaPhil ChilibeckDraško PavlovićPhilip GreinerDubravka Švob ŠtracPhilip HouckDunja DegmečićPhilip L. MarDuraiyah ThangathuraiPhilip SamDuran Ester LenoPhilip SarajlicDursun KırbaşPhumisak LouwakulDushyant DamaniaPhung Lam ToiDusica PahorPia Lopez-JornetDuyquoc NgoPia Z. Pace-AsciakDyah Wulan AnggrahiniPiazza PietroE. BonatiPicó AlfonsoE. Scott SillsPier Adelchi RuffiniEbrahim Mohammed SenanPiergiorgio DanelliEbuka E. DavidPiergiorgio FranciaEdgar Gustavo Ramos-MartínezPierre-Marie GirardEdit XhajankaPietro CacialliEdoardo BraunerPietro GrussuEdoardo MonfriniPietro ManiscalcoEdoardo PeroniPietro MazzeoEdoardo RaposioPietro MorroneEdris A.F. MahtabPilar MatudEdsaul Emilio Perez-GuerreroPing-Hsun WuEduardo De Paiva MagalhãesPin-Kuei FuEduardo SchifferPiotr AlsterEdvardas DanilaPiotr DobrowolskiEdward CatersonPiotr GabryelEdward KoifmanPiotr GawdaEfthymia PapadopoulouPiotr GronekEic Ju LimPiotr MorasiewiczEiichi WatanabePiotr RolaEiji KawamotoPiyaphong PanpisutEiko HayasePleckaityte MildaEkaterina RudnitskayaPo-Cheng HsuEkkehard SchleußnerPo-Hsuan LuEkram AliasPo-Jen HsiaoElaheh AllahyariPolašek OzrenElaheh Dalir AbdolahiniaPoonam AroraElanagan NagarajanPorter Daniel EdwardEl-Anwar Mohammad WaheedPradeep KumarElba LlopPradeep V. KadambiEleanor SeabyPradeep VaitlaEleazar Samuel Kolosovas-MachucaPraful MasteElena BernadPrahlad HoElena CancianiPrakash SahElena CsernokPranas ŠerpytisElena De-La-Barrera-ArandaPrantesh JainElena Maria ElliPrasath ManogaranElena OleaPrasenjit MondalElena S. IzmailovaPrathip PhantumvanitElena V. IndukaevaPraveen Kerala VarmaElena-Mihaela CordeanuPreeti DubeyEleni GeorgakopoulouPremashis KarEleonora LaiPriscilla SandersonEleonora Maria TreccaPrzemysław DudaEleonora NicolaiPrzemyslaw KosinskiEleonora PetrucciPrzemysław KrakowskiEleonóra SpekkerPrzemysław LisińskiEleuterio SánchezPrzemyslaw WolakElisa BannonePuo-Hsien LeElisa BelleiPushpa JoshiElisa CamelaPyziak-Skupień AleksandraElisa ReitanoQiangjun CaiElisa RubinoQichang MeiElisabeta AntonescuQimin HaiElisabeta BădilăQingzhe JiaElisabeth ZechendorfQinzhe WangElisabetta AntonelliQuanyi ZhaoElisabetta FerraraQudrat UllahEliya LevingerQuoc Thang PhamEliza RussuR. KocyłowskiElizabeta GjorgievskaR. RamkumarElizabeth Khadija TissinghRachel DaleElvan ÖçmenRachid Ait AddiElvira BratilaRachna KapoorElżbieta CiporaRadmila SparicEmad Gamil KhidrRadoica JokićEman MazyedRadosław ChaberEmanuele BarabinoRadoslaw JaworskiEmanuele BobbioRadosław KempińskiEmanuele GallinoroRadoslaw Marek KiedrowiczEmanuele MondaRadosław ZajdelEmel Sen-KilicRadu Andrei MogaEmil Marian Arbănași ArbănașiRadu BotezatuEmilio PiccioneRadu BotgrosEmilio RomaniniRadu Crisan-DabijaEmir BencaRadu MafteiEmma Rose McGloneRadu MineaEmmanouil GiorgakisRadu PrejbeanuEmmanouil KapetanakisRadu Razvan ScurtuEmmanuel Da RochaRadu V. CosteaEmre BektikRadzisław MierzyńskiEndrin KoniRafael EscateEneida PaulaRafael JimenezEng Tat AngRafael NeimanEnri NakayamaRafael SiqueiraEnrico AmmiratiRafaela AndolheEnrico BroccoRafal BartoszewskiEnrico Felice GherloneRafał GerymskiEnrico GallazziRafał JanuszekEnrico GiordanRafal KopanczykEnrico La PergolaRafał MoszyńskiEnrico MorettoRaffaele SerraEnrique BoldoRaffaella MassafraEnrique Gomez GomezRahul GuptaEnrique Perez-Cuadrado MartinezRahul VaidyaEnrique R. SorianoRaili MüllerEnwu LiuRaja VeerapandianEnzo IerardiRajesh GuptaEran R. HorowitzRajkumar Kottayasamy SeenivasagamErdoǧan SökmenRajmohan DharmarajErhan OkuyanRaluca Simona CostacheEric MauryRam Hari DahalErich HeckerRam SharmaEricsson Coy-BarreraRamadhani ChambusoErik AndersonRamana VakaErnesto González-MesaRamcharan Singh AngomErnesto Salomón BersuskyRamesh NagarajappaEshan KhanRami S. KantarEsmat RadmaneshRamin DaneshvarEsra HatipoğluRamiro Palazón-GarcíaEstelle LeclercRamon CleriesEszter DuczaRamón FuentesEugene HanRamos-Garcia PabloEugene KimRamune JacobsenEugene ShkolyarRamunė KalėdienėEugene Tze-Chun LauRamy AbdelnabyEugenia R. GatiatulinaRamy Abou GhaydaEugenio JannelliRan DuEuidong YeoRan XuEujene JungRandolph StoneEun Young ParkRanjeev Chrysanth PulleEunice CarrilhoRanko M. KutlešićEun-Jung KimRannakoe J. LehloenyaEva Češ̈kováRaphael BettachEva Katharina EggerRaphael CiumanEva MaranilloRaphael ThuillierEva María Martínez-JiménezRaquel Benedé UbietoEva Munck-WiklandRaquel De Cássia Dos SantosEvangelia KoukakiRareş Mircea BîrluţiuEvangelos KritsotakisRaúl Capote-PuenteEvann E. HiltRaúl Soto-CámaraEverett F. MagannRavi Shankar ReddyEvert F.S. Van VelsenRavindra A. PrabhuEvgeni BrotfainRavindra DeshpandeEvi FleischhackerRaviraj VankayalaEwa DudzińskaRazvan SocolovEwa PiotrowiczRazvigor Borislavov DarlenskiEwa W.A. GruszewskaRebecca ErschensEwelina Wawryk-GawdaRebecca N. SpencerF. Conceição SilvaRebekka DimpelFabian IlleReem HannaFabiana Gil MelgaçoRegina Okhuysen-CawleyFabiana NicitaRégulo López-CallejasFabiana NovellinoReinhard KoppFábio André SantosRekha BalakrishnanFábio Juner LanferdiniRemy DuleryFabio MiraldiRenan G. GomesFabio SantacaterinaRenata GržićFabrizio D'AcapitoRenata PecoticFabrizio GozziRenata VaraljaiFabrizio MartoraRenato BassanFacciorusso AntonioRenato CozziFahad KhanRenato J. VerdugoFaisal BasheerRene LeivaFangyao TangRené R.H. HartensuerFanny GuzmanRenee VickmanFarideh Jalali-MashayekhiRengaraj VenkateshFaris I. YahyaRenuka TunuguntlaFarnoosh SeirafianpourReto BaertschigerFarzad Taghizadeh-HesaryReza MazaheriFatemah Sadeghpour HeraviRhona O’ConnellFatemeh AshrafianRicardo A. Cartes‐VelásquezFatemeh KhatamiRicardo De MesquitaFatih DoganayRicardo Faria RibeiroFatih DurmuşoǧluRicardo Fernando ArraisFatih HatipogluRicardo J. JoseFatih YalcinRiccardo BeltramiFátima RoqueRiccardo BientinesiFatma A. MokhtarRiccardo MastroianniFatmir DragidellaRiccardo PozzanFavián TreulénRiccardo SarzaniFedele DonoRichard BrownFederica BuccinoRichard T. PensonFederica GalimbertiRiham M. FliefelFederica GramegnaRikhav P. GalaFederica MagagnoliRim BourgiFederica MaggiRima HajjoFederica MartoranaRima ŠileikienėFederico CacciapuotiRishika SakariaFederico CagnazzoRitthideach YorsaengFelipe J. AidarRittu HingoraniFélix A. UrraRiyad C. Karmy-JonesFélix Marcos TejedorRobert AncuceanuFen JiangRobert C. VerdonkFeng YuanRobert FreedmanFenglin ZhangRobert GałązkowskiFengwei ZouRobert HoerrFerah ArmutcuRobert J. LeClairFerdinando AgrestaRobert KarpińskiFerenc SiposRobert LeonardFernando Augusto Ugusto Mardiros HerbellaRobert PrillFernando GilsanzRobert SteinbachFernando MazzilliRobert Steven EsworthyFernando Ortiz CorredorRobert SusloFilidou EiriniRobert WestFilip MilanovicRobert Yung-Liang WangFilip StomaRobert ZajíčekFilip VitoševićRoberto A.S. CacciolaFilipe PortelaRoberto BellucciFilippo AucellaRoberto CirocchiFilippo BanchiniRoberto Lo GiudiceFilippo BasagniRobin WilsonFilippo ManelliRobinson Esteves Santos PiresFilomena CaccavaleRocio BarragánFitri Fareez RamliRodis D. PaparodisFitri OctavianaRodolfo MauceriFlaminia FerriRodrigo Fernando PesántezFlávio HojaijRodrigo Sandoval BoburgFlavio MilanaRodrigo Torres-CastroFlorence Julien-MarsolllierRodrigue PignelFlorian KraftRogelio Gónzalez-GónzalezFlorian ReckerRoger Carl GibsonFlorin BuradaRoger OveFortunato IacovelliRogerio Serafim ParraFotios DrakopanagiotakisRohadi Muhammad RosyidiFranca NataleRohan Bir SinghFranca R. GueriniRohit GuptaFrancesc AbellaRok GaspersicFrancesca ArezzoRoland GärtnerFrancesca CattoniRolf HempelmannFrancesca Katiana MartinoRoman LysiukFrancesca MameliRoman MoskalenkoFrancesco AgostiniRoman PolkinFrancesco BalestriRoman SmolarczykFrancesco BellinatoRoman ZahorecFrancesco BennardoRomana VulturarFrancesco BorgiaRomeo Petru DobrinFrancesco ColomboRomica CerganFrancesco De ChiaraRomina Marina SimaFrancesco Di BelloRong YinFrancesco Di GennaroRongqin KeFrancesco FerriniRong-San JiangFrancesco FischettiRonnie MooneyFrancesco GallettiRoopesh Singh GangwarFrancesco GaziaRosa Maria GerardiFrancesco InchingoloRosa María Giráldez-PérezFrancesco LonderoRosa Maria Tapia-HaroFrancesco MaffìaRosa SuadesFrancesco Maria CarranoRosanna VillaniFrancesco Maria GalassiRosario CianciFrancesco MoraRoslyn ComptonFrancesco OrziRossana IzzettiFrancesco PallottiRoxana Adriana StoicaFrancesco Paolo CalaceRoy MorelloFrancesco PiccirilloRubén Arroyo FernándezFrancesco SartiniRúben Miguel Nunes PereiraFrancesco Saverio LudovichettiRudin PistulliFrancesco SessaRui PoinhosFrancesco StiloRui TorresFrancesco TondoloRuomi GuoFrancesco ZengaRuth McKenzieFrancisco Javier Fernández CarrascoRuud G. J. MeulenbroekFrancisco Javier Herrero GutierrezRuwandi M. KariyawasamFrancisco Macedo PaschoalRuxandra Diana SinescuFrancisco Ruiz-CastillaRyan M. ChapmanFrancisco Sánchez‐BuenoRyoichi AsakaFrancisco SchlottmannRyszard TomaszewskiFrancisco Teixeira Da SilvaRytis RimdeikaFrancisco TustumiRyuta WatanabeFranco MusioS. RamkumarFranco VizeacoumarSa Ra LeeFrançois MontagneSaad HussainFrank Martin HaeckerSabina Di MatteoFrank R. ChenSabina Lachowicz-WiśniewskaFranz-Josef SchingaleSabine Sator-KatzenschlagerFrédéric VaysseSabine SteinerFrederique RetornazSabrina DemarieFujita YasukoSabrina Helmold HaitFumihiro OgawaSachit AnandFuming ZhangSahar HoushdaranG. Di LorenzoSaheem AhmadG. MaragathamSahin Taha KorayGa´bor FirneiszSaif KhanGábor Tamás SzabóSajjad AhmadGabriel Claudiu TenderSakineh HajebrahimiGabriel EstrelaSakshi RajoriaGabriela AniteiSalem Youssef MohamedGabriela De Morais Gouvêa LimaSalvatore AllossoGabriela RomanSalvatore BocchieriGabriela SouzaSalvatore IaconoGabriele CapoSamah SakerGabriele SorceSamar TharwatGabriele TumminelloSambri VittorioGaetano GalloSameera AbeyrathnaGaetano ScaramuzzoSami AkbulutGaia BarisioneSami M. Abd ElwahabGambardella ClaudioSamir SalamaGamze YetişmişSamo FokterGao HuaSamrad MehrabiGargi MahapatraSamrita DograGarry KuanSamuel DontaGaryfallia PeperaSamuel R. FernandesGassan MoadySamuel X. ShiGatot PurwotoSamuele VaccariGaurav DhawanSamy M. RiadGautham GampaSana MajidGauthier LoronSanam DolatiGee Jun TyeSandeep Kumar MishraGeetu BhandoriaSandeep ThannaGelei XiaoSandra CecconiGemma Caterina Maria RossiSandra RodriguesGennaro Mariano LenatoSandra TorresGennaro PerroneSandro PasqualiGeorg GelbeneggerSanja Popovic-GrleGeorge BazoukisSanja Zoričić CvekGeorge Calin DindeleganSanthanam AmuthaGeorge FotakopoulosSanthosh RajamaniGeorge H. ThompsonSantiago J. BallazGeorge L. HinesSantiago Navarro LedesmaGeorge R. KasyanSantiago Ortiz-PerezGeorge SamanidisSantino Ottavio TomasiGeorge UmemotoSantos CastañedaGeorges MjaessSantos FernandoGeorgi As. GeorgievSantosh S. JeevannavarGeorgia NtaliSara De CarolisGeorgios D. PanosSara PariniGeorgios KatsarasSara RegnérGeorgios LaliotisSara WawrysiukGeorgios Pappas-GogosSarah R. T. SeidelGeorgios Sokratis ChatzopoulosSaranya VaradarajanGerardo PellegrinoSarath VijayakumarGerd Marie AleniusSarchielli PaolaGergő JózsaSareena Hanim HamzahGerhard HaidlSarfaraz IqbalGerhard NahlerSarfaraz K. NiaziGermain HonvoSari MattilaGerold HolzerSarika GuptaGhasem BaratiSarina Levy-MendelovichGhasem FarahmandSaritphat OrrapinGhassan MaaloufSasa RajsicGheorghita IsvoranuSatilmis BilginGiacomo AndreaniSatish VishwanathaiahGiacomo CasaliniSatoru OnizukaGiacomo FarìSatoshi IshikoGiacomo PapottoSatoshi KomasaGiada BianchettiSatoshi YanagisawaGiamberto CasiniSaurabh ChandanGiampietro GubbiniSaurabh ChaturvediGian Andrea PelliccioniSaurabh JainGian Luca MarellaSaurabh LuthraGian Luigi GigliSaverio MuscoliGianCarlo PecorariSavvas LampridisGiandomenico BisacciaScerrino GregorioGianfilippo CaggiariSebastian BöttgerGianluca CasseseSebastian Garcia-ZamoraGianluca CiardiSebastian JäckleGianluca CiolliSebastian LotzienGianluca FaddaSebastian MertowskiGianluca GaidanoSebastian SchnaubeltGianluca GambariniSebastian WawrockiGianluigi PastaSébastien SchmerberGianmaria D'AddazioSedighe KarimzadehGianpaolo ZerbiniSeetharaman HariharanGică NicolaeSegundo Nicolas SeclenGilad RegevSelami Aykut TemizGina GheorgheSelim BozkurtGinés HernándezSelvaraj GurudeebanGinszt MichałSema K. AydedeGIORDANO AlessandraSenija EminovićGiorgia AilunoSenne BroekxGiorgio LofreseSenthilkumar SankararamanGiorgio MantovaniSeogsong JeongGiovana TorrezanSeokchan EunGiovanna PiccoloSeong-Hee Maria KoGiovanni CangelosiSeow Hui TeoGiovanni CannarozzoSepideh OmidvariGiovanni CochettiŞerban Mihai BǎlǎnescuGiovanni CrisafulliSerbülent TürkGiovanni CristalliSergey GurevichGiovanni Dell’Aversana OrabonaSerghei CovantevGiovanni MatricaliSergio CortelazzoGiovanni MigliareseSergio De SalvatoreGiovanni MonniSergio FacchiniGiovanni PapaSergio GarciaGiovanni SerioSergio L. Stevan, Jr.Ģirts BriģisSergio MoralGiudice AmerigoSergio RecaldeGiulia DallagiacomaSerkan YílmazGiulia Di DalmaziSeth TarrantGiulia Di StefanoSeverin LäuchliGiulia GibiinoSevgül DönmezGiulia PurpuraSeyed Ahmad Naseri-AlaviGiuliana ScarpatiSeyed Alireza DastgheibGiuliano MarchettiSeyed Arash AlawiGiulio VaraSeyed Massood NabaviGiuseppe BenagianoSeyed Mohammad Ali Hosseini RadGiuseppe CelentanoSeyed Mohammad AyyoubzadehGiuseppe CilibertiShaghayegh Baradaran GhavamiGiuseppe LiscoShah-Hwa ChouGiuseppe LosurdoShahriar ShahiGiuseppe MaccagnanoShai FactorGiuseppe MandraffinoShaimaa HusseinGiuseppe MasciaShakeel IjazGiuseppe RivaShalini DograGiuseppe Roberto GiammalvaShang-Ju WuGiuseppinella MelitaShankar Jaikishan EvaniGladiol ZenunajShankargouda PatilGlauco Akelinghton Freire VitielloShanmuga S. MahalingamGlenda Giorgia CaputoShanwen ChenGloria González-MedinaSharat ChandraGo AnanShashank ShekharGonçalo DiasShaun SabicoGonzález-Pérez RupertoShehwaz AnwarGonzalo Navarro-FernándezSheila BermejoGonzalo Solís SánchezShelly R. McFarlaneGoran KoračevićSheng ZhuGrażyna DykowskaSheng-Long DingGrażyna Jarza̧bek-BieleckaSherouk FoudaGrażyna NowickaShibili NuhmaniGrażyna SygitowiczShigeaki NakazawaGrażyna Wyszyńska-PawelecShigenaga MatsuiGreggi TizianaShigeru NakaiGregor StavrouShijie HeGregory J. TsayShingen NakamuraGroener DanielShing-Jye ChenGrzegorz CieslarShingo KuwataGrzegorz JakubiakShingo TsujinakaGrzegorz KoniecznyShin-ichi KonnoGrzegorz M. KozeraShinnichi OkazumiGrzegorz MichalskiShintaro FujiharaGrzegorz RabaShintaro HorieGrzegorz StarobratShinya KitamuraGuanjun YangShinya MatsuzakiGuglielmo ManentiShinya TomariGuglielmo ManticaShinyu IzumiGuglielmo Niccolò PiozziShipra AgarwalGuilherme Grossi Lopes CançadoShirley HillGuillaume GiraudShiroma Mangalika HandunnettiGuillermo GrazioliShishir Ram ShettyGuinevere GraniteSho YamakawaGülen Yerlikaya-SchattenShobana NavaneethabalakrishnanGulseren AkyuzShoenfeld YehudaGulzhanat AimagambetovaShohei KawaguchiGündüz YümünShoshi KeisariGuoli DaiShuaiying CuiGürdal SahinShubha Ranjan DuttaGuru MaitiShu-Hua YangGustavo Desire Antunes GastalShujiro HayashiGustavo PimentelShumaila Aman TasneemGustavo Tadeu VolpatoShung-Te KaoGvozden RosicShunsuke KudoGwenllian TawyShuya KandoriGyan ChandShu-Yi WangHabib Md Reazaul KarimShyikuen WuHadeel HalawehSiavash RaiganiHafiz Muzzammel RehmanSiddharth DugarHaidy AbbasSiddharth NarayananHaishu LinSigbjørn BerentsenHaitham AbdelhakimSile A. CreedonHaiwen LiSilverio RotondiHaiyan YangSilvia Alberti-ViolettiHaizhou ZhuSilvia De Miguel-BilbaoHalil Ibrahim TanriverdiSilvia FilippiHallie ThomasSilvia LeonciniHamayak S. SisakianSilvia MonacoHamdi ChtourouSilvia PalmaHamid PakmaneshSilvia RiondinoHamid Reza AbbasiSilvia SpotoHamid Reza KatoozianSimin SharifiHamoon ZohdiSimon DublerHana OrlíkováSimon SchoechlinHani AlhadramiSimona BungauHani Amir AouissiSimona Catalina StefanHanna LiberskaSimona CimaHans MickleySimona MitevaHans WolfSimona ZaamiHans-Christian SiebertSimona-Roxana GeorgescuHans-Oliver RennekampffSimone ChiolaHantai KimSimone La PadulaHanvedes DaovisanSimone MorraHany GabraSimone MorselliHao TangSimone PaliniHaralds PlaudisSimone RedaelliHarold L. RekateSimone SavastanoHarshani WijerathneSimonida TomićHarun AchmadSina RashediHarvinder Singh ChhabraSinescu CosminHasan NaveedSinisa BabovicHashimoto KenjiSinjini SikdarHasnaa JalouSiqi HuHassan RashidiSirajudheen AnwarHassan RasouliSirish DharmapuriHayato TerayamaSiu Hong WongHazem M. MousaSiv-Elin Leirvaag CarlsenHazir RahmanSlađana MilardovićHe LiuSlavoljub ŽivkovićHeather ClelandSławomir Jan TeperHebah A.L. UbeedSławomir WoźniakHeber Arbildo-VegaSlim SmaouiHeike HelmholzSlobodan SpasojevicHeike KielsteinSlobodanka Lemajic-KomazecHeitor Andrade JrSmriti PandaHeitor O. SantosSnehal RautHelen FogartySnezana LukicHelen YifterSnezana P. PolovinaHelena FelgueirasSnježana TomićHelena JoséSo Yeon Joyce KongHelena MartynowiczSoedarsono SoedarsonoHelene Santos BarbosaSofia SaraivaHelge StalsbergSoghra BagheriHemendra GhimireSohail SyedHeming YaoSokol TrunguHenglong HuSomnath PandeyHenrik C. BäckerSonam KumariHenrik FalhammarSonglin PengHenrik Max JensenSonia FantoneHenrique SilvaSonia NathHenry LiuSonja JäckleHenry PleassSoojie K. YuHenry SutantoSophia LetsiouHerko GrubitzschSoraya PazHeura Llaquet-BayoSorin HostiucHideki KadoneSorin J. BrullHideki MiyachiSorin Tiberiu AlexandrescuHidemi NakataŠoša IvanHidemichi KouzuSotirios SotiriouHideo FujitaSouhail HermassiHideo HashizumeSoultana MeditskouHideo SuzukiSoumojit PalHidetaka NomaSouvagya PanigrahiHideyuki KinoshitaSowndramalingam SankaralingamHideyuki MaedaSpyridon SimantirisHideyuki YoshitomiSrdjan B. AleksandrićHikichi YutaSrdjan D. PoštićHinne A. RakhorstSreelakshmi PanginikkodHiroaki KobayashiSridhar MuthusamiHiroaki TobaSriram VaidyanathanHirokazu InoueSrjit DasHiroki TeragawaSruthi RamagiriHiroki YokotaStamatia TheodoridouHironori IshiguchiStanislav KotlyarovHiroshi ArakawaStavros TryfonHiroshi FurukawaStefan Cristian VesaHiroshi TanahashiStefan HertlingHirotaka NakashimaStefan Paul-AndreiHiroyuki HiraiStefan WeberHisanori IkumaStefania CimbanassiHisham FansaŞtefania TudoracheHitendra Singh SolankiStefano BallestriHitoshi MaruyamaStefano CanosaHoang Van ThuanStefano De ServiHoejeong ChungStefano FuscoHojong ChoiStefano GennaiHolyavka Marina G.Stefano GonnelliHong Jin KimStefano MagnoHongtao JinStefano Marco Paolo RossiHongyang XuStefano Maria PriolaHoracio Matías CastroStefano MariniHorng Chi-TingStefano MenzoHortensia Moreno-MacíasStefano PalermiHridaya ShresthaStefano RauseiHrvoje MihaljStefano TuroloHsiang Ning LukSteffen GrautoffHsin-An ChangŠtěpán HavránekHsin-Chung ChengStephan LindemannHsu Wen-LiStephan PayrHsuan Yu ChenStephan VogtHsu-Heng YenStephanie JarvisHuaitian LiuStephanie Rodríguez BesteiroHuapyong KangStephen J. UsalaHuarong LuStephen JenningsHugh GashStephen WallaceHugo M. BotelhoSteven L. WarsofHugo RamosSteven LevyHugues GeorgesSteven M. ParnesHui HuangSteven Paul NisticòHui Yin LimSteven SilversteinHui-Rong JiangStoyan KostovHuitzilihuitl Saucedo-OrozcoSubendu SarkarHung ChangSubhi YousifHung-Chang LeeSubramanyam DasariHung-Pei TsaiSuchada KongkiatkamonHung-Pin TuSudhahar VaradarajanHurriyet YilmazSuenghwan JoHussain TahzibaSueyoshi MoritaniHussam AliSugasini DhavamaniHwa-jung KimSuhaila Abd MuidHye Chang RhimSu-Ho LimHysi DorjanSukanya SahaHyun-Chang LimŞükrü ÇağlarHyung-Il KimSukru Serdar BalciHyungju KwonSunao TokumaruHyunil LeeSundus JavedI.D.F.S.F. BoinSung Eun KimIb JammerSung Huang Laurent TsaiIbrahim E. EfeSung Soo AhnIbrahim El-BattrawySungbae MoonIbrahim M. SayedSung-Ho HerIda AringerSung-Hyoun ChoIdda Maria LauraSunghyun PaickIgnazio StanganelliSung-kyu KimIgor DumicSung-Sahn LeeIgor KhatkovSunil KarhadkarIgor KomnikSunil S. KarhadkarIgor KovačevićSunita PatelIizuka TakashiSupin ChompoopongIkchan JeonSupriya GuptaIkhwan RinaldiSurajit BasakIlaha IsaliSuresh K. VermaIlan MerdlerSusan ClarkeIlaria BenzoniSusan M. WebbIlaria CampioniSusan Motch PerrineIlaria SammarraSusana García-SilvaIlham Wan MokhtarSusanne Rospleszczİlknur Sur ErdemSusmita BarmanIlona DjoricSuveenkrishna PothuruImam AmmarullahSuzy WeemsIman TavassolySven PischkeImed LatiriSven VetterImena RexhepiSvenja SydorImtiyaz Ahmad WaniSvetislav TatićIndu G. RajapakshaSvetlana M. Vrzić-PetronijevićInemesit Okon BenSvetlin TsonevInes Ben GhezalaSvetozár DluholuckýInés Llamas-RamosSwagata Arvind TambéInes MonteSwati AnandInga-Lena NilssonSyed Ahmad Chan BukhariIngunn DybedalSyed NabilInmaculada BanegasSyed Tahir Abbas ShahInti ZlobecSyed Thouheed AhmedIoanna C. KoniariSylwia Dzięgielewska-GęsiakIoannis A. GiantsisSylwia SmarzewskaIoannis AnagnostopoulosSzczepan PiszczatowskiIoannis BoletisSzymon SkoczynskiIoannis LiampasTabito KinoIoannis MaroulisTadamitsu MatsudaIoannis PantazopoulosTadashi NakamuraIoannis PetrakisTadatsugu MorimotoIoannis TsamesidisTadeusz AmbrozyIoannis VamvakarisTae Han KimIoannis VasileiadisTae Hyun BanIoannis VlastosTae-Hwan KimIraia Muñoa HoyosTahir NooraniIram MurtazaTahmina Nasrin PolyIrena GlažarTaia Maria Berto RezendeIrena HocevarTaishi HataIrena MladenovaTakafumi YokotaIrene KarampelaTakaharu OueIrene MadrigalTakahiro HiraideIrene Torres-SánchezTakahiro NoharaIrfan KhanTakamasa KinoshitaIride Francesca CeresaTakane Kikuchi-UedaIrina BalázsTakanori SaitoIrina Magdalena DumitruTakao ShimazoeIrina TicaTakashi MatsuzakiIrmina MorawskaTakashi NuriIsabel Cláudia Masson Poiares BaptistaTakashi OginoIsabel Poiares BaptistaTakayuki MasakiIsabel SalvatTakayuki SaitohIsabella PisaniTakehiro IwataI-Shiang TzengTakeshi OgawaIslam MohamedTakeshi YamasakiIsmaheel O. LawalTakeshi Yamashitaİsmaıl AytacTaku OshimaIsmini LasithiotakiTakuma InagawaIsrael Pérez-TorresTakumi InamiIssam KoleilatTamal SadhukhanIssam SalibaTamara GulicIssam TanoubiTamás BaranyaiIstván Ferenc ÈdesTancu Ana Maria CristinaIstván OsztheimerTanıl KendirliIulia-Cristina BagiuTanja Grubić KezeleIuliana NenuTanya J. LehkyIvan BudimirTao YangIvan CukTara HenaganIvan FistonićTaro IkegamiIván Herrera-PecoTatiana AntipovaIvan KopitovićTatiana Sella TunisIvan LandiresTatiana SidiropoulouIvan PuljizTatjana GoranovičIvan SabolTatsuya AbéIvan Y. IourovTatsuya ImabayashiIvana GrgićTawfik KhouryIvana Hanzec MarkovićTawin InpankaewIvana JochmanováTazzioli GiovanniIvana KavecanTeodora Alexa-StratulatIvana NovakTepetes KonstantinosIvana NovakovicTerenzio CosioIvana P. NedeljkovicTeresa CatalanoIvana SutejTeresa GianiIvanka DimovaTeresa LettiniIvo Dumic-CuleTeresa PerraIvo SoaresTeresa Rubio-TomásIvonne NelTeruhiko ImamuraIwan August MeynaarTetiana SkrypetsIwona Homa-MlakThadiyan Parambil IjinuIwona Poziomkowska-GęsickaThemistokles GrigoriadisIwona SzydłowskaTheodore C. HannahIwona WertelTheresa DuelloIzabela WinklerThiago Pavoni Gomes ChagasIzabela ZakrockaThiemo DingerJ. Patrick O'ConnorThirumal RajJ.U. ChinchuThomas BouchardJ.W. GuoThomas HankJacek SmerekaThomas J. GastJacek SzczygielskiThomas M. TiefenboeckJacek WilczyńskiThomas PerreaultJack Bee ChookThomas RückschloßJacob E. TeitelbaumTianyu LiJacob GopasTihomir MustakovJacobo Rodríguez-SanzTim WalkerJacobus MullerTimotius Ivan HariyantoJacobus Van Der VeldenTina B. McKayJae Hoo LeeTing HanJae Yon WonTito BrambulloJaeho ChungTiuri E. KroeseJae-Hwi NhoTiziana CiarambinoJae-Sung YooTiziano TestoriJae-Wook ChungTobias M. BallhauseJae-Young LeeTobias PiegelerJagoš GolubovićTobias RufJahanpour AlipourTolga OlmezJai Young ChoTom KovitwanichkanontJaideep MahendraTom P. WalshJaideep SandhuTomas CarrollJaime DevineTomáš KrejčíJaime Vásquez-GómezTomas NovotnyJakub KornackiTomás TeodoroJakub RuszowskiTomas ZarembaJakub SzabelskiTomasz BlicharskiJames H. WynneTomasz CudejkoJames HallinanTomasz FrączykJames M. ClearyTomasz HirnleJames RowleyTomasz LitwinJames W. McKowenTomasz MarjanskiJames WilliamsTomasz RechcińskiJamir Pitton RissardoTomasz TokarekJan Adriaan GrawTomasz WandtkeJan BieńkiewiczTomasz ZąbekJan BilskiTomasz ZdrojewskiJan GrosekTomasz ZubilewiczJan KotarskiTomislav KokicJan TrnaTomislav KuzmanJan UlrychTommaso ErcoliJana MašlankováTommaso GrecoJana OsackaTommaso Roberti Di SarsinaJanaki DeepakTomofumi NishinoJanelle R. Noel-MacDonnellTomoki NakamuraJanez RebolTomomitsu MiyagakiJanis GardovskisTomotaka SaitoJanja TrčekTomoyuki HiokiJanko SamardzicToni ModricJansons JurisToni PollinJanusz R. WłodarczykToshiharu FujiiJanusz SielskiToti LucaJapneet KaurToyokawa TatsuyaJaromir AstlTraian DumitrașcuJarosław DulskiTrifan Anca-VictoritaJarosław PinkasTsai-Ming LinJaroslaw RegułaTsoi HoJasmina BobanTsung-Hsien LeeJasna JančićTsung-Jen HuangJason MitchellTsuyoshi OhtaJason YuenTudor Sorin PopJavaid NaumanTutku SoyerJavier MartínTyng Yuan JangJavier Quillo-OlveraUdo RolleJavier. CampaniniUdo SeedorfJaw-Yuan WangUenis TannuriJay S. MishraUgo MarcheseJayakumar NairUgur CanpolatJean François D. CadranelUiju ChoJean R. HughesUlderico FreoJean-Louis PrudhonUlf BoekelerJean-Marc RetrouveyUlises De La Cruz-MossoJeena BansalUlrich GlitschJeff VictoroffUlrich OrdaJefferson ChenUlrich W. PreussJegarubee BavananthasivamUmair MuhammadJelena AntićUmberto MoscatoJelena KosticUmesh Tharehalli MathadaJelena PrpicUrmi KhannaJelena RoganovicUrszula Sajewicz-RadtkeJelena ViderUwe Von FritschenJennifer HunterVadim V. GenkelJennifer M. VojtechVahid RakhshanJennifer Van WykVaibhav A. DixitJenn-Ming YangValentin Nicolae VarlasJen-Pin ChuangValentin SinitsynJenq-Shyong ChanValentina ChiariniJens Rolighed LarsenValentina IzzoJeong HeoValentina LupatoJeremy LevyValentina Oana BudaJernej StareValentina TostoJérôme AddaValentina TsibizovaJérôme CosteValeria CorradettiJerzy Franciszek KozielskiValeria LuzziJesse C. KrakauerValeria MussoJesse HatgisValeria StatiJesse RumboValeria ViscoJessica A. WrightValerio MaisJessica IorioValery V. LikhvantsevJesús Martínez De La CalValeska HofmannJesús Molina-MulaValliappan MuthuJeyasakthy SaniasiayaVan Giau VoJi LiuVandilson Pinheiro RodriguesJiajia XiVanessa BeutgenJian ZhouVanessa Faria CortesJianlei GuVânia GomesJiawen YongVanita Pathak-RayJie ChenVasile DrugJie ZhuVasile V. BintintanJien Jiun ChenVasileios PapaliagkasJien-Jiun ChenVasiliki ChantziaraJim DavieVasiliki MollakiJimena MuciñoVasiliki TsigkouJin BaiVasilios KotsisJin WangVasudev BallalJin ZhangVasudeva IyerJing PangVasudevan RamachandranJingping WangVenkatesh KatariJin-Hee LeeVenketesh SivaramakrishnanJinhu XiongVenturella RobertaJinn-Rung KuoVeronica Alonso-ArroyoJinwei ZhangVeronica AranJiong MeiVerónica F. Morán-BarrosoJiong ZhouVeronica MartiniJiří MoláčekVeronica MercuțJiri PlasekVerónica Rocío Vásquez-GarzónJiro KishimotoVeronika HuntošováJitendra KanshanaVesa Cosmin MihaiJitendtra Kumar Singh PariharVesna I. KesićJiun-Lin YanVesna VucicJiun-Wen GuoVibhav GautamJoan BagóVibhu Krishnan ViswanathanJoanna BartosińskaVicky VargheseJoanna BoniorVíctor García-GonzálezJoanna FilipowskaVictor InfanteJoanna StefanowiczVíctor M. RiveraJoanne Van RynVictor MartinJoão BotelhoVida DemarinJoão Gustavo Oliveira ClaudinoVidya RattanJoão M. SantosViet TranJoaquim Murray Bustorff-SilvaVignesh ShanmugamJoaquin Manzo-MerinoVijaya Anand ArumugamJoaquín SopenaVikas JhawatJochen D. SchipkeVikash AgrawalJochen SchaeferVikash JaiswalJochen WilhelmViktor ReshetnikovJoel EllwangerVilai KuptniratsaikulJohan Vandee WalleVilallonga RamonJohanna FearyVilma DudonieneJohannes GrevenVinay GoyalJohannes M. AlbesVincent PonsJohannes WachVincenzo CandelaJohn A. Albert St. CyrVincenzo CataneseJohn A. BernatVincenzo De RosaJohn GriniatsosVincenzo GiordanoJohn K. YueVincenzo NuzziJohn Mackay SøftelandVincenzo Ostilio PalmieriJohn O. OnukwuforVincenzo PatellaJohn OngVincenzo QuagliarielloJohn SlavinVincenzo ZanonJohn T. SchulzVinod VijayakurupJohn W. BolngaViola SalvestriniJohnatan Torres-TorresVioleta DediuJohn-David AubertVirginia CasiglianiJo-Hsuan WuVirginia Durán Muñoz-CruzadoJolita BadarieneVirginia NavarroJonas A. PramuditaVirginie GuastellaJonas Michel WolfVirginie Monnet-CortiJonathan BayuoVitalii LunovJonathan D. HoVito PesceJonathan LuisiVitor FerreiraJonghwa JangVittoria PerrottiJonhatan SilvaVittorio DibelloJonnathan Guadalupe Santillán-BenítezVittorio LippiJoon Hyuk ChoiVivek AnandJoon Kee LeeVivek PandeyJoost BierensViviana MucciJoost RutgesVladiana TuriJorg BahmVladimir A. ShvartzJörg PieperVladimir BilimJőrg VienkenVladimír MusilJorge Góngora-RodríguezVladimir SobolevJorge MartinsVladimir VimbergJorge Moreno CharcoVlasta DostalovaJorge N.R. MartinsVlasta FesslovaJorge Ordonez LlanosVolker Martin TronnierJorge R. Fernández-SantosVrinda GoteJorge Velázquez SaornilVui King Vincent-ChongJose A. O. SimoesVyacheslav OgayJosé Antonio MingoranceVytaute PeciulieneJose BaezaWalentyna BalwierzJosé Bañuls-RocaWalicyranison Silva-RochaJosé Bernardo Noronha MatosWalid D. FakhouriJose Boix-OchoaWalter L. StrohmaierJosé EleutérioWalther BildJosé Fernandes NetoWang Peng-YuanJosé Francisco López-GilWang XiaogangJose Garcia De La TorreWang ZhengJosé López-CastroWanguo LiuJosé Luis Clúa EspunyWanich SuksatanJosé Luis Pardal-RefoyoWanni LinJosé Luis Pérez-CastrillónWaqar A. QureshiJosé Luis Vázquez NogueraWasan KatipJosé M. Palacios-JaraquemadaWassan NoriJosé M. PobleteWataru IsonoJosé Manuel López GarciaWayne BerryhillJosé Maria LassoWei ChengJose Maria Pereira De GodoyWei XiongJosé Miguel Seguí-RipollWei-chi WuJosé Valentín García GutiérrezWeijia LiJose-Angel Perez-RiveraWei-Ju ChangJosefa Gonzalez-SantosWeiqian WangJosefa LeónWei-Wei DengJosé-María Sánchez-GonzálezWei-Yi WuJosep Lluís Clua-EspunyWeiyu JiangJoseph P. Hornak Wen-Cheng LoJoseph S. AlpertWen-Chih LiuJosé-Tomás NavarroWenjing ZhangJoshua DenhamWenzhen YuanJosip A. BorovacWerner CordierJosip VrdoljakWeronika GonciarzJosko BozicWeronika RymerJosue MartosWieland HermannJothi VargheseWiesław BłachJovana JuloskiWieslaw ZiolkowskiJowy Yi Hoong SeahWietse J. EshuisJuan Antonio Martínez-TadeoWilliam BelangeroJuan Manuel Guzmán-FloresWilliam D. WatsonJuan Miguel Martínez-GalianoWilliam Mark ErwinJuan Mozas-MorenoWilliam Y. RaynorJuan Valdés-StauberWilmer Gianfranco Silva-CasoJuan-Francisco Peña-CardellesWinston A. MorganJudit SymmankWisit KaewputJudy E. SternWitold StrebJuhani H. MäättäWitold TłustochowiczJulia E. Marquez-ArricoWi-Young SoJulia LischkaWojciech SkrobotJulian TangWojciech WolanśkiJulie GueudryWolfgang A. WetschJulie SwartzendruberWolfgang SenkerJulieanne P. SeesWolfram Trudo KnoefelJūlija VoicehovskaWon JungJulio Calleja-GonzalezWon-Bae ParkJun Jie Benjamin SengWoo-Jong KimJunaid AhmedWook Jin ChoiJune-Sung KimWouter L.W. Van HemertJung Hee KimWu Po-TingJung-Sub AnXia HaoJungtae LeeXiang WuJunhel DalanonXianghao ZhanJun-ichi TanumaXiaodong BaoJunji ShiodeXiaofeng LiuJunying LiXiaohua LiuJustin BeilbyXiaojian ShiJustina MotiejūnaitėXiaoju HuJustus Patrick BeierXiaoling ZhouJustyna ZińczukXiaolong LiJuxian SongXiaoxiao SunJyoti KumarXiaoyan WangK. Thomas RobbinsXiaoyu MaKah Kheng GohXinghua WangKai ShenXinlei LiKai-Chieh ChanXiuhua WengKaisa Leea SunelaXiuying ZhaoKai-Wen HsuXochitl A. Ortiz-LeónKalliopi PlatoniXu ZhangKalpana NagpalXueyu HuKalyana ChakravarthyY.C. LeeKamariah IbrahimYaichiro OkuzuKamel LaghmaniYang LiKamil KoszelaYang YangKamila RachubińskaYang-Gun SuhKamilla StachYanjie ZhangKanako NoritakeYanmin WanKandaswamy EswarYao-Ching HungKannan SridharanYashdeep PhanseKanokporn PinyopornpanishYashima KazuoKanta KidoYasser E. ShaheinKaori UedaYasser Musa IbrahimKaoruko ShimizuYasuhiro TakahashiKara B. AndersonYasuki HoriKara HollowayYasunori TomeKarel AllegaertYasunori YamashitaKarel KostevYasushi NishiiKarel LábrYasushi TsujiKaren P. AckerYau Kei ChanKarl-Heinz WidmerYehoshua ShapiraKarol RycerzYeliz PriorKarolina ChilickaYen-Fu ChengKarolina KossakowskaYeou-Guang TsayKarsten Henning WredeYerim KimKarthik RagunathanYi Chiang HsuKarthik RamasamyYi FanKarthikeyan MuthusamyYi ManKaschina ElenaYi SunKassiani TheodorakiYifei ZhangKatarína BabinskaYihao LinKatarina BaralićYi-Hsing ChenKatariná BobockáYi-Hsuan HsiaoKatarzyna HojanYing Bin YanKatarzyna HolcmanYing SunKatarzyna Komosinska-VassevYitayal Addis AlemayehuKatarzyna MadziarskaYI-TING LINKatarzyna Mocny-PachońskaYi-Wen ChiuKatarzyna Ogrodzka-CiechanowiczYi-Wen WangKatarzyna Plagens-RotmanYogesh MarfatiaKatarzyna SobierajskaYohan KerbageKatarzyna SzkudelskaYohei HayashiKatarzyna WalkiewiczYoichi SakuradaKaterina GrafanakiYoji KishiKaterina KambouriYong ZhanKaterina NikolitchYongchel AhnKaterina ProkopiouYonghui HuangKatharina K. RallYongjae YooKatharina RumpYong-Ming YuKatherine WoodYongseok JeeKathrin Bohn-WippertYoon-Seob KimKathrin WelckerYoshifumi IkedaKathryn Y. BurgeYoshihiro FukumotoKatia BarbaroYoshihiro IkuraKatsuya KitamuraYoshihiro KamadaKavita SinghYoshihiro TakamuraKazuhiro MoriyamaYoshihisa KotaniKazumichi FujiokaYoshikane YamauchiKazunori NagasakaYoshikazu KishinoKazuya InokiYoshiko KidaKazuyoshi ImaizumiYoshinori InagakiKazuyuki HirookaYoshiro HoraiKazuyuki MatsushitaYouness El BakriKegan Romelle JonesYoung Eun LeeKeiichi HosakaYoung Koo LeeKeming GaoYoung Suk KwonKeng-Fu HsuYousef Ahmed FouadKenji HayashidaYu KoyamaKenji KayashimaYuanchuan ZhangKenneth A. Kenneth A. ArndtYubbu PutriKennio PaimYu-Ching ChouKent NakamotoYuhki SatoKentaro OkazakiYuichi NishiokaKeshen LiYuichi SaitoKeskanya SubbalekhaYuichiro YahataKeun Ho LeeYuji ShimizuKevin ChenYuki HagaKevin HackshawYuko HiguchiKevin MartellYukoh OharaKeyur VoraYuksel AltinelKhai Pang LeongYuli FradkinKhajornsak TragoolpuaYu-Lung HsuKhaldoon AljerianYun HuangKhaled El MatriYun Jin KimKhaled HarrarYune ZhaoKhaled MusallamYung-Shun JuanKhalid Omer AlfaroukYung-Shuo KaoKhalil A. KhatriYunqi TangKhalim KhujamatovYunsong LiuKhizar HayatYun-Wen ChenKi-cheong ParkYurii ChepurnyiKielan Darcy McAlindenYuriy Vasil'evKiichi HirotaYusra Habib KhanKi-Il LeeYusuke NakagawaKim An NguyenYutang WangKimberly A. KewYu-Wei LiuKin Israel NotarteZachary Mitchel BaumanKiran Kumar GanjiZahra AshenaKiran Kumar SoniZaki HakamiKirsten WittkeZbigniew AdamiakKishore BalasubramanianZbigniew RaszewskiKit-Aun TanZehuan LiaoKiyofumi TakabatakeZeinab GhazanfariKiyohide IshihataŽeljko AntićKlaudia DopytalskaZenon PogorelićKlearchos PsychogiosZhao WangKlemen GrabljevecZhaowei KongKoh KitagawaZhaozhong ZhuKohei OkuyamaZhen QiuKoji EbisumotoZheng Zachory WeiKoji OtaniZhenghan WangKoki KosamiZhengwu PengKondapa Naidu BobbaZheng-Zheng ZhangKonlawij TrongtrakulZhihao ChenKonosuke NakajiZhiping LiuKonrad Leszek MendralaZhongkai WangKonstantin MuranovZhongwei LiuKonstantin TziridisZiad Said DahdouhKonstantinos A. GatzoulisŽiga KozincKonstantinos G. KyriakoulisZikai HaoKonstantinos PapadopoulosZilvinas DambrauskasKonstantinos StergiosZixin WangKonstantinos TriantafyllouZizy Ibrahim ElbialyKosho YamanouchiZlatko KljajicKosuke OtaniZoard T. KrasznaiKosuke YoshidaZoe FlorouKouji NagataZohaib YousafKoulaouzidis AnastasiosZoran KarlovićKoulla ParpaZornitsa Ivanova ZlatarovaKovy Arteaga-LiviasZsolt SarszegiKoya OzawaZsuzsanna RecsanKrešimir CrnogaćaZubair AhmadKrishna Kishore UmapathiZulvikar Syambani UlhaqKrishna M. SundarZuzana GdovinovaKrishnan GanapathyZuzanna RzepkaKrister Lindmark



